# Comparative Study of the Molecular Basis of Pathogenicity of *M. bovis* Strains in a Mouse Model

**DOI:** 10.3390/ijms20010005

**Published:** 2018-12-20

**Authors:** Guangyu Cheng, Tariq Hussain, Naveed Sabir, Jiamin Ni, Miaoxuan Li, Deming Zhao, Xiangmei Zhou

**Affiliations:** State Key Laboratories for Agrobiotechnology, Key Laboratory of Animal Epidemiology and Zoonosis, Ministry of Agriculture, National Animal Transmissible Spongiform Encephalopathy Laboratory, College of Veterinary Medicine, China Agricultural University, Beijing 100193, China; hnchdd@163.com (G.C.); drtariq@aup.edu.pk (T.H.); naveedsabir@upr.edu.pk (N.S.); jiamin1014@cau.edu.cn (J.N.); limx1102@cau.edu.cn (M.L.); zhaodm@cau.edu.cn (D.Z.)

**Keywords:** *M. bovis*, immune response, pathogenicity, virulence factors, mutation

## Abstract

It is widely accepted that different strains of *Mycobacterium tuberculosis* have variable degrees of pathogenicity and induce different immune responses in infected hosts. Similarly, different strains of *Mycobacterium bovis* have been identified but there is a lack of information regarding the degree of pathogenicity of these strains and their ability to provoke host immune responses. Therefore, in the current study, we used a mouse model to evaluate various factors involved in the severity of disease progression and the induction of immune responses by two strains of *M. bovis* isolated from cattle. Mice were infected with both strains of *M. bovis* at different colony-forming unit (CFU) via inhalation. Gross and histological findings revealed more severe lesions in the lung and spleen of mice infected with *M. bovis* N strain than those infected with *M. bovis* C68004 strain. In addition, high levels of interferon-γ (IFN-γ), interleukin-17 (IL-17), and IL-22 production were observed in the serum samples of mice infected with *M. bovis* N strain. Comparative genomic analysis showed the existence of 750 single nucleotide polymorphisms and 145 small insertions/deletions between the two strains. After matching with the Virulence Factors Database, mutations were found in 29 genes, which relate to 17 virulence factors. Moreover, we found an increased number of virulent factors in *M. bovis* N strain as compared to *M. bovis* C68004 strain. Taken together, our data reveal that variation in the level of pathogenicity is due to the mutation in the virulence factors of *M. bovis* N strain. Therefore, a better understanding of the mechanisms of mutation in the virulence factors will ultimately contribute to the development of new strategies for the control of *M. bovis* infection.

## 1. Introduction

Tuberculosis (TB) remains one of the most important infectious diseases of humans and animals worldwide. The disease is caused by *Mycobacterium tuberculosis* complex (MTBC), especially, *Mycobacterium tuberculosis* (*M. tuberculosis*) and *Mycobacterium bovis* (*M. bovis*). It is reported that 10.4 million new cases and 1.4 million deaths were caused by TB in 2015, and 2.8% of new cases of tuberculosis are attributed to *M. bovis* infection [1,2]. Like many other developing countries, control of bovine tuberculosis remains a challenge in China, which is mainly caused by *M. bovis*. 

Tuberculosis consists of multiple stages that depend on both the pathogen and the host immune response to the infection [3,4]. Innate immune responses play an important role in bacterial control. There is increasing evidence that genetic variation among mycobacterial isolates contributes to differences in immune response induction and disease progression [5,6,7,8]. However, variability in the innate immune responses to genetic differences among *M. bovis* strains remains poorly understood. A better understanding of the genetic factors involved in immune response to infection will contribute to the control of bovine tuberculosis. 

Since the first genomic study of *M. tuberculosis in* 1998, many additional studies have shown the genomic details of MTBC strains [9,10]. Though various strains of MTBC exhibit a high degree of genetic conservation, even then they exhibit a variable degree of pathogenicity [11,12,13]. This variation in the pathogenicity of MTBC strains is probably caused by the diversity in genetic and evolutionary background [14,15]. Therefore, it is necessary to understand the role of genetic diversity of different pathogenic strains of *M. bovis* in disease development.

In this study, we investigated the severity of disease in mice infected with *M. bovis* C68004 strain and *M. bovis* N strain. *Mycobacterium bovis* N strain was isolated from brain tissue of infected cattle with typical signs of systemic tuberculosis. Based on the clinical and histopathological observations in mice, we hypothesized that this strain of *M. bovis* may have distinct virulence factors and would show different pathogenesis in mice compared with the *M. bovis* C68004 strain. 

As the natural route of infection of *M. bovis* is inhalation, we compared these two *M. bovis* strains with different genetic and evolutionary background using an intranasal infection mouse model [16,17]. We observed substantial variation in the disease progression and cytokine expression profiles in mice infected with two different strains of *M. bovis*, which showed altered degreesof pathogenesis. We further compared the whole-genome sequence (WGS) of both strains to identify the virulence factors that may contribute in the pathogenesis of these two strains.

We found that mice infected with *M. bovis* N strain showed acute death and severe inflammatory signs than those infected with *M. bovis* C68004 strain. The multiplex cytokine analysis assay for the serum samples of mice infected with *M. bovis* N strain also resulted in an increased production of IFN-γ, IL-17, and IL-22 in comparison to *M. bovis* C68004 strain. Comparative genomic analysis showed 750 single nucleotide polymorphisms (SNPs) and 145 small insertions/deletions (InDels) between the two *M. bovis* strains. After matching with the Virulence Factors Database (VFDB) database [18], we found mutations in 29 genes, which relate to 17 virulence factors. Expression of these genes was mainly related to the secretion system and cell surface transport. Out of 29 mutated genes, nine were present in C68004 while 20 existed in the N strain. Overall, our results demonstrate that mutations in the virulence factors contribute to the variation in the pathogenicity of *M. bovis* strains. The identification of mutated virulence factors in the pathogenic strains of *M. bovis* lead toward a better understanding of disease progression and further studies are suggested to develop new strategies for the control of *M. bovis* infection.

## 2. Materials and Methods 

### 2.1. Ethics Statement

All animal experiments were performed according to the Chinese Regulations of Laboratory Animals − The Guidelines for the Care of Laboratory Animals (Ministry of Science and Technology of People’s Republic of China) and Laboratory Animal Requirements of Environment and Housing Facilities (GB 14925±2010, National Laboratory Animal Standardization Technical Committee).

The animal studies and research protocols were approved by The Laboratory Animal Ethical Committee of China Agricultural University under the license number cau20170108-2 (8 January, 2017).

### 2.2. Mice and M. bovis Strains

Female BALB/C mice aged between 6–8 weeks were obtained from specific-pathogen-free facilities at Charles River Laboratories and were maintained in BSL3 facilities. 

In the present study, we used two virulent strains of *M. bovis*. *Mycobacterium bovis* C68004 strain was obtained from the China Institute of Veterinary Drug Control and this strain has been used for years with stable virulence. The *M. bovis* N strain was isolated recently from the brain tissue of cattle with generalized bovine tuberculosis (Figure S1). These strains were identified as *M. bovis* by multilocus sequence typing analysis as described before [19]. The *M. bovis* N strain was isolated in our laboratory and only cultured for few passages, while the other strain was obtained from cold storage and was passaged for three times after re-isolated from experimentally infected animals. Both strains were grown to mid-log phase in Middlebrook 7H9 broth (BD Biosciences) supplemented with 10% *v*/*v* OADC (Oleic acid, Albumin, Dextrose, Catalase) enrichment medium (BD Biosciences) and Tween 80. Before animals were infected with *M. bovis* strains, we calculated the colony-forming unit (CFU) by obtaining the optical density (OD) value for each isolate.

### 2.3. Genomic DNA Extraction, Sequencing, and Analysis

Genomic DNA from the two *M. bovis* strains were extracted using a TIANamp Bacterial Genomic DNA Kit (Tiangen Biotech Co. Ltd., Beijing, China), and were sequenced on an Illumina HiSeq 2000 platform. Raw sequencing reads were aligned to the reference genome *Mycobacterium bovis* AF2122/97 (LT708304.1) using the bwa mem alignment algorithm version 0.7.12 with default settings [20]. Single nucleotide polymorphisms (SNPs) and insertion/deletion (INDELs) events were identified using the Genome Analysis Tool Kit Haplotype Caller [21]. After combining variant call files for both strains, SNPs were filtered with the setting QD < 3.0 || FS > 60.0 || MQ < 40.0 || MQRankSum < −12.5 || ReadPosRankSum < −8.0 and INDELs were filtered with QD < 3.0 || FS > 200.0 || ReadPosRankSum < −20.0 as recommended in software documentation. Variants common to both isolates were then removed leaving only mutations variable among strains *M. bovis* C68004 and *M. bovis* N. Variants were annotated according to the reference genome gene annotations using snpEff version 4.3t [22]. Large deletions are difficult to assess using GATK, so we used the PILON genome analysis tool to look for additional larger deletion sequences [23].

### 2.4. Bacterial Infection

Mice (*n* = 6 per group) were anesthetized by Zoletil 50 (Virbac, France), which was first diluted 5-fold and 50-μL per mouse (0.5mg) was injected intraperitoneally. The BALB/c mice were divided into fifteen groups, and each group contain six mice. The animals were challenged via intranasal route with 200, 500, 1000, 2000, 5000, 10,000 and 20,000 CFU of *M. bovis* C68004 or *M. bovis* N diluted in phosphate buffer saline (PBS) according to the protocol described elsewhere [16]. The inoculum size was quantified after 24 h of infection by determining CFU in the lung tissue of 3 animals after infection with 200 CFU for both isolates and we found that the animals were successfully infected with *M. bovis* strains. We observed mean 159 ± 19 CFU in animals infected with *M. bovis* N strain while 172 ± 31 CFU in the lung tissue of animals infected with *M. bovis* C68004 strain. The control group of mice was challenged with PBS. All mice were maintained in the cages fitted with micro-isolators. Mice health was controlled by daily checking for smoothness of the fur, movement and activity, and reaction to the external stimuli. All of the mice inoculated with 10,000 and 20,000 CFU of both strains contracted acute death around the fourth week post inoculation. Therefore, we selected the animal groups infected with 5000 CFU for the long-term survival experiment. We observed a clear difference in the body weight of mice infected with 2000 CFU of *M. bovis* N showing a weight loss of 15% or more around four weeks post-inoculation as compared to the control group. In addition, previous studies showed that bacterial replication in the lung, spleen, and liver of mice challenged with the low dose of *M. bovis* reached a plateau at four weeks post infection [16,24,25]. Therefore, we sacrificed all the 2000 CFU and below infected mice by day 30 post-inoculation. Similar results were observed from two independent experiments.

### 2.5. Histopathology 

All the mice infected with a CFU of 2000 or below were weighed and sacrificed on day 30 post-inoculation. We referred to a previous study and developed a clinical score method to calculate the severity of disease progression [26]. The mice were scored in terms of general behavior, feeding habits, and weight gain or loss. Animals reaching a clinical score of ≥4.0 were at the time point that followed by the time of death. The weight of each lung and spleen was measured, and organ coefficient was obtained by using the following formula: organ coefficient = organ weight (g)/mouse weight (g) [27]. Blood was obtained ante-mortem from eye route and serum was collected after clotting. The blood serum samples were stored at −80 °C until further experiment. The same lobe of the lung from each study group was fixed in 4% phosphate-buffered formaldehyde and embedded in paraffin. Sections of ~5 μm thickness were prepared and stained with haematoxylin and eosin (H&E), and with Ziehl-Neelsen (ZN) for histological examination and for the detection of acid-fast bacilli, respectively [28]. The digital images of the whole slide were acquired by Hamamatsu NanoZoomer S60 slide scanner and its viewing platform (NDP Viewer). 

### 2.6. Multiplex Cytokine Assay

The concentrations of various cytokines and chemokines in blood serum were determined by ProcartaPlex^TM^ Multiplex Immunoassay (eBioscience, San Diego, CA, USA) according to the manufacturer instructions, which contains 18 cytokines (GM-CSF, IFN-γ, IL-β, TNF-α, IL-1β, IL-2, IL-4, IL-5, IL-6, IL-9, IL-10, IL-12 P70, IL-13, IL-17, IL-18, IL-22, IL-23, and IL-27). We preferably used multiplex immunoassay for the determination of various cytokines and chemokines because of the limited quantity of serum samples from each animal [29,30].

### 2.7. Statistical Analysis

Results are presented as mean and SEM (standard error) within each group (*n* = 6). For analysis of organ coefficients, clinical score, and cytokine production, we used student’s unpaired *t*-test (two-tailed). Data were analyzed using the SPSS Statistics version 20.0 (IBM, Armonk, NY, USA), the ImageJ version 1.45 (National Institutes of Health, Bethesda, MA, USA) and the GraphPad Prism version 5.01 program (GraphPad Software Inc., San Diego, CA, USA). A *p* < 0.05 was considered as statistically significant. The *p*-values shown in the figures are presented with *** (*p* < 0.001), ** (*p* < 0.01), * (*p* < 0.05), and n.s. indicates statistically non-significant.

## 3. Results

### 3.1. Clinical Presentation and Necropsy Lesions

**Figure 1:**
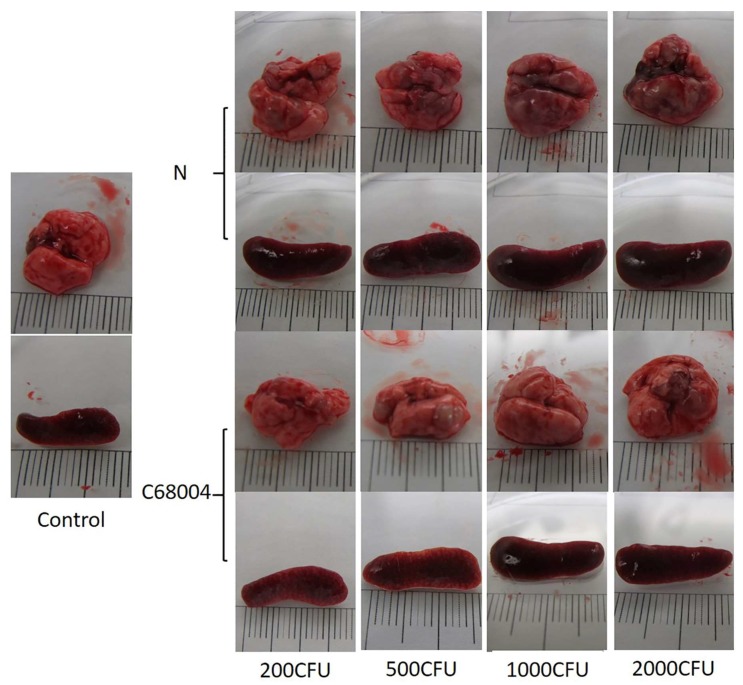
Representative photographs showing gross lesions of the lung and spleen of mice infected with different strains of *Mycobacterium bovis*. The lung and spleen were aseptically removed from the mice at the end of the clinical observation (30 days post-inoculation) before death. Pictures of the lung and spleen of mice infected with 200, 500, 1000, and 2000 colony-forming unit (CFU) of *M. bovis* N and *M. bovis* C68004 strains are shown individually. Control shows the lung of the mice inoculated with phosphate buffer saline (PBS).

The infected animals showed clinical signs around 19 days post-inoculation. The animals infected with *M. bovis* N with 5000 CFU and above showed obvious weight loss, disordered hair, somnolence, inappetence, and conjunctivitis. The animals infected with 2000 CFU of *M. bovis* N showed similar signs when sacrificed on day 30 post-inoculation, while no visible signs were observed in animals infected with the same CFU of *M. bovis* C68004 strain (Figure S2). On gross examination of organs, we observed that the longitudinal size of the lung and spleen were 2–5 millimeters longer in the infected groups as compared with the control group. Normal tissue could barely be seen in the lung of *M. bovis* N strain infected animals while several nodular lesions were present in *M. bovis* C68004 infected animals (Figure 1). Overall, the mice infected with *M. bovis* N showed more severe organ lesions and symptoms of the disease than *M. bovis* C68004. Similar results were observed in two independent experiments.

### 3.2. Survival Time

**Figure 2:**
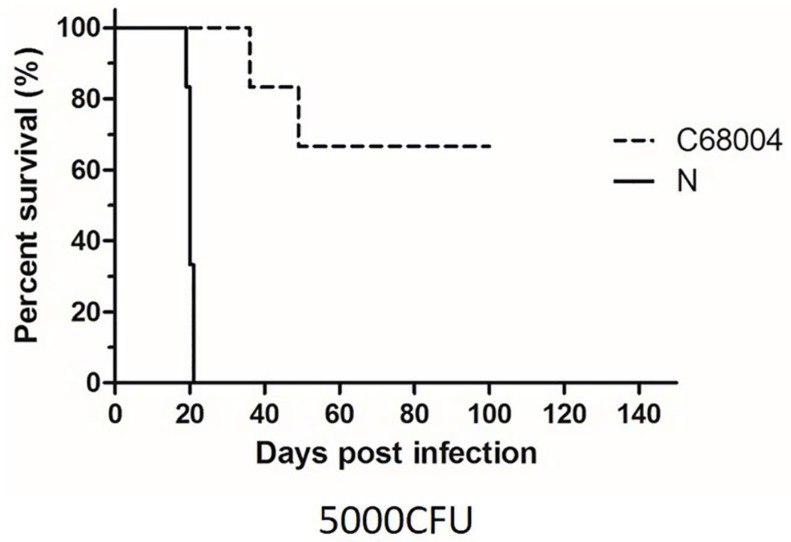
The survival rates of mice infected with 5000 CFU of *M. bovis* N and *M. bovis* C68004 strains. Each group had six mice. Observation times (day post-inoculation) are indicated in the *x*-axis. The experiment was repeated two times independently.

To assess the difference of pathogenesis between the two *M. bovis* strains, we also observed the survival time of mice post infection [31]. We evaluated the survival time for mice infected with 5000 CFU of both strains. A dose of 5000 CFU of *M. bovis* N strain resulted in acute death (median survival time 20–22 days post challenge). The mice showed clinical symptoms around 19 days post-inoculation and acute death happened in three days. On the other hand, mice infected with 5000 CFU of *M. bovis* C68004 survived significantly longer than those infected with *M. bovis* N, only two mice died around 40 days post-inoculation (Figure 2). The animal survival experiment was also repeated twice and similar results were obtained.

### 3.3. Organ Coefficient and Clinical Score of Mice Infected with Different Strains of M. bovis

**Figure 3:**
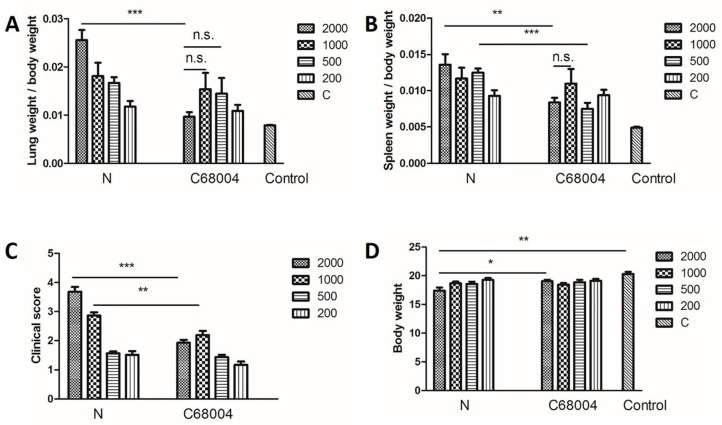
Organ coefficient of the lung (**A**) and spleen (**B**), score of clinical symptoms (**C**) and body weight (**D**) from mice infected with different CFU of *M. bovis* N and *M. bovis* C68004 strains or PBS 30 days post-inoculation. The experiment was repeated two times independently. Statistical analysis was performed using student’s unpaired *t*-test (two-tailed). Bars represent means SEM (*n* = 6); * *p* < 0.05, ** *p* < 0.01; *** *p* < 0.001.

In order to evaluate the effect of *M. bovis* infection on lung and spleen, we measured the weight of these organs. We observed that the lung and spleen of mice infected with either strain of *M. bovis* with a CFU of 2000 and below were heavier as compared with the uninfected control group. The organ coefficient of *M. bovis* N infected mice showed that the weight of lung and spleen was positively correlated with the dose of infection. Moreover, the organ coefficient also demonstrated that lung and spleen of mice infected with 500 and 2000 CFU of *M. bovis* N strain were heavier than of those infected with *M. bovis* C68004 (Figure 3A,B). On the other hand, no significant difference was observed in the weight of lung and spleen of mice infected with *M. bovis* N and *M. bovis* C68004 with 200 and 1000 CFU. The lung and spleen of mice challenged with 2000 CFU seemed lighter than the groups receiving lower doses but no significant difference was observed between 2000 CFU and below CFU infected mice with *M. bovis* C68004 strain. The mice were monitored for clinical signs of disease on a daily basis as described in Materials and Methods section. The clinical symptoms were more severe in mice infected with 2000 and 1000 CFU of *M. bovis* N than of those infected with *M. bovis* C68004 strain, as reflected by the clinical score (Figure 3C). By comparing with the uninfected mice, the significant body weight loss was only observed in mice infected with 2000 CFU of *M. bovis* N (Figure 3D). Similar results were observed in two independent experiments.

### 3.4. Histopathological Examination

**Figure 4:**
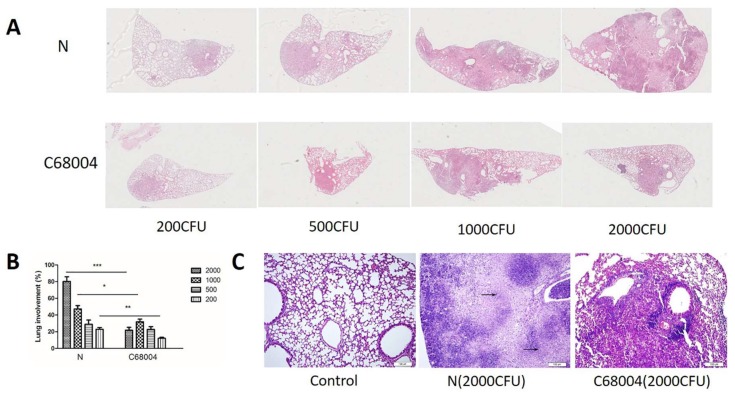
Development of lung lesions in mice infected with *M. bovis* N and *M. bovis* C68004 strains. The lung and spleen were aseptically removed from the mice at the end of the clinical observation (30 days post-inoculation) before death. (**A**) The whole-slide digital images of H&E-stained lung sections. Pictures of the lung of mice infected with 200, 500, 1000, and 2000 CFU of the both strains are shown individually. (**B**) Morphometric analysis demonstrated affected lungs in mice treated with the both *M. bovis* strains. Results are represented as percentage of lung surface area involved, calculated using ImageJ viewer software (obtained from six animals per group). (**C**) Granulomatous lesions in lung infected with 2000 CFU of the both strains. Pictures of the lung of mice infected with 2000 CFU of *M. bovis* N and *M. bovis* C68004 strains and uninfected control are shown individually. The arrows indicate multiple necrotic areas in the lung of mice infected with 2000 CFU of *M. bovis* N (arrows: necrosis). Scale bar: 100 μm. * *p* < 0.05, ** *p* < 0.01; *** *p* < 0.001.

**Figure 5:**
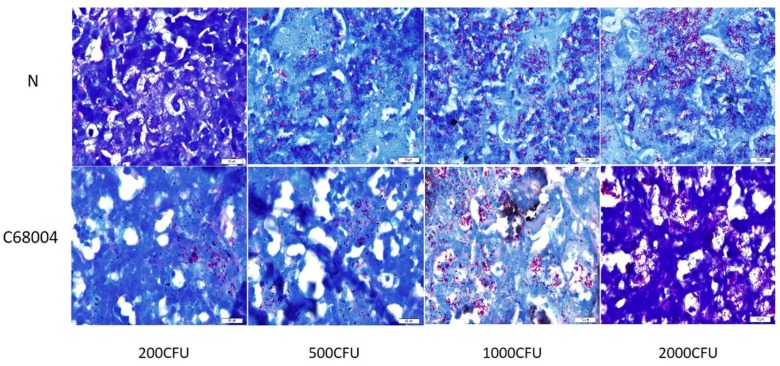
Acid fast bacilli in infected lung. High magnification of central necrotic areas showing *M. bovis* bacilli within cellular debris of infected lung. Scale bar: 10 μm.

For histopathological examination, the lung tissues of infected and un-infected control mice were stained using H&E staining and were observed under light microscope. We found multiple dispersed inflammatory areas in the lung tissue of all animals infected with 2000 CFU and below. We observed clear difference of the lesions in the lung of mice infected with 1000 CFU and 2000 CFU of the two strains and larger inflammatory areas were seen in lung of mice infected with *M. bovis* N as compared with *M. bovis* C68004 (Figure 4A). The percentage of lung surface area inflammation had a statistically significant increase in *M. bovis* N treated mice as compared with *M. bovis* C68004 (Figure 4B). All the inflammatory areas were infiltrated with lymphocytes and macrophages in all infected animals compared with control animals (Figure 4C). In addition, we observed several inflammatory regions with multiple necrotic foci in the lung of animals infected with high CFU of *M. bovis* N strain than those infected with *M. bovis* C68004 strain (Figure 4C). In addition, we observed visible acid-fast bacilli in the lung sections of all infected mice showing that infection occurred in all group of mice at different administered doses for both strains of *M. bovis* (Figure 5). These experiments were repeated twice and found similar results for histological changes in the lung tissue. 

### 3.5. Cytokine Profiles in Serum

**Figure 6:**
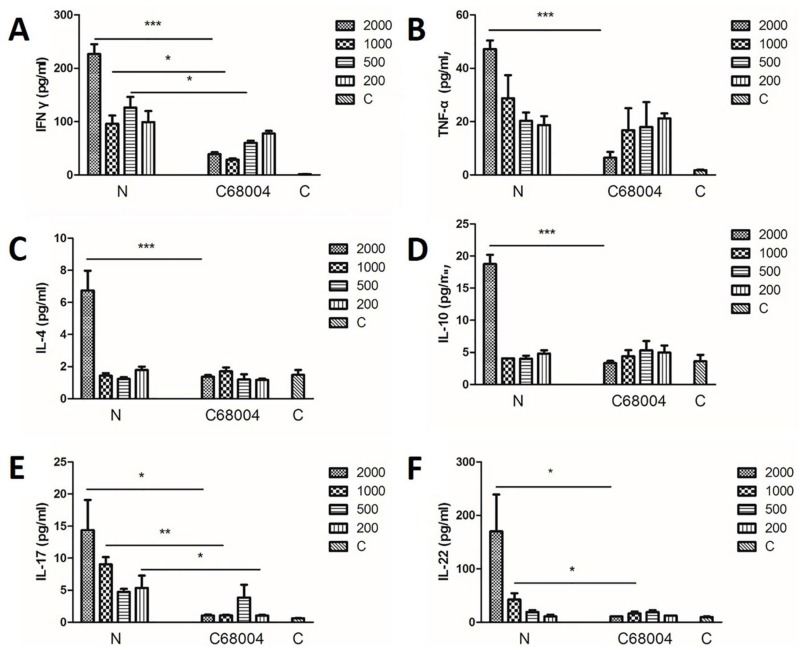
Cytokines expression profiles in mice infected with different CFU of *M. bovis* N and *M. bovis* C68004 strains. The blood was harvested ante-mortem via eye route and serum was used for cytokines expression analysis. Th1 immune response cytokines shown are IFN-γ (**A**) and TNF-α (**B**). Th2 immune response cytokines shown are IL-4 (**C**) and IL-10 (**D**). Th17 immune response cytokines shown are IL-17 (**E**) and IL-22 (**F**). Statistical analysis was performed using student’s unpaired *t*-test (two-tailed). Bars represent means SEM (*n* = 6); * *p* < 0.05, ** *p* < 0.01; *** *p* < 0.001.

In order to investigate immune responses induced by the both *M. bovis* strains, we assessed the level of various cytokines in the serum samples. The cytokine analysis was done by multiplex cytokine assay. We found significant difference in cytokines (IFN-γ, IL-4, IL-10, IL-17, IL-22, and TNF-α) expression in all infected animals that have roles in the regulation of T cell responses including T helper1 (Th1), T helper2 (Th2), and T helper17 (Th17) (Figure 6). While no significant difference was observed for the remaining cytokines among various infected animal groups (Figure S3). The level of IFN-γ in the mice infected with *M. bovis* N was significantly increased when compared with the *M. bovis* C68004 infected mice, except the animals challenged with 200 CFU for both strains (Figure 6A). *Mycobacterium bovis* N strain also induced higher levels of IL-17 production in mice than *M. bovis* C68004 infected mice, except for the mice challenged with 500 CFU (Figure 6E). In addition, we observed an increased level of IL-22 only in mice infected with 2000 CFU and 1000 CFU of *M. bovis* N than *M. bovis* C68004 strain infected mice, while no significant difference was recorded in the animal groups infected with CFU of 200 and 500 of both strains of *M. bovis* (Figure 6F). Similarly, the production of TNF-α, IL-4, and IL-10 was only significantly high in the *M. bovis* N exposed group when the animals were infected with 2000 CFU (Figure 6B–D). These results show that an increased inflammatory response was observed in mice infected with *M. bovis* N strain as compared with *M. bovis* C68004 strain.

### 3.6. Genomic Sequence Analysis

Multilocus sequence typing (MLST) analysis confirmed that both strains were members of *M. bovis*, so we next performed whole-genome sequencing (WGS) to identify genetic differences that may explain the observed differences in pathology and immune responses induction between these isolates. Comparing both isolates with the reference *M. bovis* strain AF2122 (ATCC##), we identified 750 single nucleotide polymorphisms (SNPs) and 145 small insertions/deletions (InDels) that differentiated the two isolates (Table 1, Supplementary Materials Tables S1 and S2). Of these, 467 SNPs and 86 InDels happened in *M. bovis* N strain and the rest existed in *M. bovis* C68004 strain. In addition, 115 SNPs and 55 InDels belonged to the PPE/PE-PGRS gene families [32]. 

### 3.7. Virulence Factors Mutation in Both Strains

We then searched for virulence factors by comparing these changes against 257 virulence factors collected from the VFDB database [18]. We found that 20 genes were mutated in *M. bovis* N strain and nine genes were mutated in *M. bovis* C68004 strain in comparison to the reference strain. The results showed that 17 virulence factors were variable between the two strains of *M. bovis* (Table 2). These data unveiled that higher level of mutations was found in *M. bovis* N strain in comparison to C68004 strain and these mutations in the virulence factors seems to be responsible for the high degree of pathogenicity of the N strain in the mouse model of infection. 

## 4. Discussion

Bovine tuberculosis represents a significant animal health problem having economic impact and also imposing zoonotic threat to human health [2,33]. It is estimated that the prevalence of bovine tuberculosis is 9% worldwide, especially in the developing countries [24]. Therefore, it is important to study the factors related to pathogenicity of different *M. bovis* strains, which could help in the early diagnostic procedure and adopting new strategies for the control of the disease. Although many studies have been reported on the difference in virulence of *M. tuberculosis* strains, only a few studies provided information on the variation in virulence among different strains of *M. bovis* [33,34,35,36]. In the current study, we analyzed the genetic differences of two *M. bovis* strains in mouse models that were isolated from cattle with different backgrounds.

To date, BALB/c and C57Bl/6 mice are the most classical tuberculosis resistant animal models and are widely used for *M. tuberculosis* or *M. bovis* studies, although both of them show limited features of tuberculosis pathophysiology when compared with the C3HeB/FeJ mouse model [24,37,38]. Based on previous studies and experience in our laboratory, the BALB/c mouse model is the best choice for *M. bovis* infection through the nostril [16,39]. In our current study, we infected the animals with different CFUs to determine the suitable dose of both *M. bovis* strains that cause clinical disease. Therefore, we infected the animals with various CFU starting from 200 to maximal 20000 CFU. We found that the animals exposed to 10000 CFU and above with both strains of *M. bovis* died acutely around four weeks post infection (data not shown). Based on these observations we focused on animal groups infected with 200 to 5000 CFU to analyze the pathogenicity difference between two *M. bovis* isolates in our study. We found that all animals had died after three weeks of infection with 5000 CFU of *M. bovis* N strain. This finding of early-mortality of mice is similar to a previous study in C3HeB/Fej mice infected with *M. tuberculosis* [24]. On the other hand, animals challenged with *M. bovis* C68004 strain survived for much longer time post-inoculation. It showed that both strains of *M. bovis* were not similar regarding their pathogenicity level in mice. In a previous study, BABL/C mice inoculated with more than 1000 CFU virulent *M. bovis* strain via lateral tail vein suffered from acute death with a median survival time of 22 days [16]. Previous studies on non-human models of mycobacterial infection suggest that the key period to determine whether the mice will show the progression of acute or chronic infection is between 3–6 weeks post infection [40,41]. Consistent with that, we found that the mice infected with the highest dose of *M. bovis* N showed an acute onset of infection while the animals infected with *M. bovis* C68004 developed progressive chronic infection. Animals infected with lower CFU than 5000 showed clinical symptoms after three weeks of infection. When the mice were sacrificed on day 30 post-inoculation, we found a variable number of small and large tubercles in the lung of all infected animals. In addition, the lung and the spleen were also enlarged in mice exposed with both strains of *M. bovis* compared with the control uninfected mice. Like the reference strain *M. bovis* AF2122 (ATCC##), both of the strains caused inflammatory response areas in BABL/c mice [24]. Considering the discrepancy of the organ coefficient, clinical scores, and the gross lesion examination, *M. bovis* N may cause a more severe inflammatory reaction in lung and spleen than *M. bovis* C68004. Based on the findings of acid-fast staining of lung sections for all infected mice, we found significantly more *M. bovis* bacilli in mice infected with 2000 CFU of *M. bovis* N strain than mice infected with *M. bovis* C68004. The above findings suggest that *M. bovis* N strain is more pathogenic compared to *M. bovis* C68004 strain.

This conclusion was supported by further analysis of cytokine levels in the serum samples of mice infected with both strain of *M. bovis*. Cytokines are critical for recruitment and activation of immune modulating cells to the site of infection and are essential for the control of bacterial growth and survival [42]. Both IFN-γ and TNF-α have been accepted as the most important Th1 cells cytokines playing a key role in the progress and outcome of tuberculosis infection [43,44]. IFN-γ has been considered as a potential biomarker for the identification of various stages of *M. tuberculosis* infection [45,46]. Recent studies revealed that IFN-γ and IL-17 play an important role in pro-inflammatory responses and chemokine regulation, and a stronger IFN-γ and IL-17 response correlates with the severity of the disease in humans [44,47]. In vitro and in vivo generation of Th17 cells can lead to stronger IL-17 and IL-22 expression [48]. Our finding showed that *M. bovis* N strain induced stronger IFN-γ, IL-17, and IL-22 responses than *M. bovis* C68004 strain. In line with the histopathological observation, we considered stronger IFN-γ, IL-17, and IL-22 responses in mice induced by different *M. bovis* strains may be related with the severity of the disease. TNF-α is a protective cytokine and most virulent mycobacterial strains elicit an increased expression of TNF-α than avirulent mycobacterial strains [49,50]. In our study, we only found significant difference of TNF-α response in the 2000 CFU level of infection between the two strains, same as other cytokines including IL-4, IL-10, GM-CSF, IL-1β, IL-2, IL-6, IL-13, and IL-18. It is generally accepted that the outcome of mycobacterial infection depends on the induction of host immune responses but unrestricted immune responses are deleterious and may lead to the disease exacerbation [51,52]. Therefore, this could be interpreted as chaotic immune responses when the mice infected with 2000 CFU of *M. bovis* N were close to death. In addition, we found no significant difference in the expression of TNF-α, IL-4, and IL-10 induced by two different strains of *M. bovis*. Unexpectedly, in our study, we found significantly low levels of IFN-γ in animals infected with high CFU (1000 to 2000) of *M. bovis* C68004 strain than animals infected with low CFU of the same strain. This may be due to faster activation of adaptive immunity in the higher doses of *M. bovis* C68004 compared with lower doses.

The increasing availability of WGS data for *M. tuberculosis* has shown that the evolution of *M. tuberculosis* strains contributes to lineage-specific adaptation of human populations, disease pathogenesis, and drug resistance [13,53]. That is why tuberculosis remains one of the leading causes of death worldwide. We hypothesized that the epidemic of *M. bovis* strains nowadays may have stronger pathogenesis and better bovine adaptation. Therefore, we investigated the genome difference between the two strains for further understanding of variation in the degree of pathogenicity. Upon WGS analysis for both strains of *M. bovis*, we found many SNPs and InDels between these two strains that may account for the observed differences in pathology and immune responses. After the comparison with the reference virulence factors collected from the VFDB, there were 17 virulence factors related to 29 genes in the two strains. Of these, nine existed in *M. bovis* C68004 strain and 20 existed in *M. bovis* Nstrain. The proteins expressed by these genes are mainly involved in secretion and cell surface transport, like trehalose-recycling ABC transporter and phthiocerol dimycocerosates (PDIMs) and phenolic glycolipids (PGLs). Acquisition of host sugars is quite important during some stages of *M. tuberculosis* infection, trehalose-recycling ABC transporter is highly specific for uptake of the disaccharide trehalose and essential for virulence of *M. tuberculosis* [54]. Phthiocerol dimycocerosates (PDIMs) and phenolic glycolipids (PGLs) are structurally-related complex lipids in the mycobacterial cell wall and present only in pathogenic mycobacteria, such as *M. tuberculosis* and *M. bovis* [55]. The loss of PDIMs and PGLs has been associated with decrease in virulence of *M. bovis*, although the contribution of individual PDIMs and PGLs to virulence in these organisms has not been evaluated [56]. In addition, some studies have focused on the mutations in mice of these genes and resulted virulence variation. For example, mutation in mice operons, *espG1* gene, and *ppsD* gene may attenuate *M. tuberculosis* virulence [57,58,59]. However, in our study, the mutations of PDIMs, PGLs-related genes, and the above three genes happened in *M. bovis* N strain. We suggest that the mutations in these genes may not change the gene function. After all, most studies have focused on the relation between the loss of the virulence factors or related genes and the virulence change in *M. tuberculosis*. It is necessary for us to identify these virulence factors in *M. bovis* and find out what variants play key roles for the different clinical presentation in mice. 

According to the limited reports, cattle maintain both *M. bovis* and *M. tuberculosis* in China and the infection with non-tuberculosis mycobacterium was also reported recently [60,61]. The *M. bovis* N strain has more genome differences compared with the reference *M. bovis* strain AF2122 (ATCC##) than the *M. bovis* C68004 strain. In addition, considering its strong pathogenicity, it may have well developed bovine adaptation, can aggravate the problems in bovine tuberculosis eradication, and might have a potential zoonotic risk to human in China. 

Taken together, the data demonstrate that both strains of *M. bovis* are diverse in terms of intracellular adaptation and the progression of disease. In addition, the data elucidate that different strains of *M. bovis* induce a varied level of immune responses in the infected animals. Genomic-based analysis reveals several mutations in the virulence factors responsible for the degree of pathogenicity. The study widens our knowledge of *M. bovis* strains associated virulence factors, and the possible role of these factors in pathogenicity and determining the outcomes of the infection.

## Figures and Tables

**Table 1:** Mutation type of single nucleotide polymorphisms (SNPs) and insertions/deletions (InDels).

SNP		InDel	
Intergenic region	86	Conservative inframe deletion	13
Missense variant	409	Disruptive inframe insertion	26
Stop gained	7	Frameshift variant	60
Stop lost & splice region variant	2	Frameshift variant & stop gained	4
Synonymous variant	246	Intergenic region	31
		Pilon deletion	11

**Table 2:** Related genes of *M. bovis* C68004 and N strains.

Gene Mb#	VFclass	Virulence factors	Strain C68004	Strain N
Mb0607	Mammalian cell entry (mce) operons	Mce2	-	mce2D
Mb1214	Cell surface components	Glycopeptidolipid (GPL) locus	-	papA3
Mb3856	Cell surface components	Trehalose-recycling ATP-binding cassette (ABC) transporter	fad23	-
Mb3906	Secretion system	ESAT-6 secretion system (ESX)-1 (T7SS)	espI	-
Mb3916c-Mb3917c	Secretion system	ESX-2 (T7SS)	-	mycP2
	Secretion system	ESX-2 (T7SS)	-	eccD2
Mb3924C	Secretion system	ESX-2 (T7SS)	eccC2	-
Mb0208C	Metal uptake	Heme uptake	-	mmpL11
Mb0250C	Cell surface components	GPL locus	-	fadE5
Mb0661C	Cell surface components	Methyltransferase	mmaA4	-
Mb0994	Metal exporters	Copper exporter	-	ctpV
Mb1193	Anaerobic respiration	Nitrate reductase	-	narG
Mb1267	Cell surface components	Trehalose-recycling ABC transporter	-	lpqY
Mb1553C	Cell surface components	GPL locus	-	gtf1
Mb1766C	Anaerobic respiration	Nitrate/nitrite transporter	narK2	-
Mb2139C	Proteases	Proteasome-associated proteins	mpa	-
Mb2404C	Metal uptake	Mycobactin	-	mbtB
Mb2439C	Secreted proteins	Enhanced intracellular survival protein	-	eis
Mb2958	Cell surface components	Phthiocerol dimycocerosate (PDIM) and Phenolic glycolipid (PGL) biosynthesis and transport	-	ppsC
Mb2959	Cell surface components	PDIM and PGL biosynthesis and transport	-	ppsD
Mb2960	Cell surface components	PDIM and PGL biosynthesis and transport	-	ppsE
Mb2966	Cell surface components	PDIM and PGL biosynthesis and transport	-	fadD28
Mb2982C	Cell surface components	PDIM and PGL biosynthesis and transport	-	-
Mb3298	Metal exporters	P(1)-type Mn2+ transporting ATPase	-	ctpC
Mb3631C	Lipid and fatty acid metabolism	Pantothenate synthesis	panD	-
Mb3851	Cell surface components	Trehalose-recycling ABC transporter	sap	-
Mb3896	Regulation	RegX3	espG1	-
Mb3912C	Secretion system	ESX-1 (T7SS)	-	eccE1
Mb0606	mce operons	Mce2	-	mce2C
Mb0609	mce operons	Mce2	-	mce2E

## Supplementary Materials

The supplementary materials are available online at http://www.mdpi.com/1422-0067/20/1/5/s1.
