# Supplementary material for: Comparative Study of the Molecular Basis of Pathogenicity of M. bovis Strains in a Mouse Model
Source: Int J Mol Sci. 2018 Dec 20;20(1):5. doi: 10.3390/ijms20010005 (PMC6337294; doi:10.3390/ijms20010005)
Supplement: Supplementary file 1 [file ijms-20-00005-s001.zip › ijms-395434 Supplementary materials/Supplementary material.docx]

**
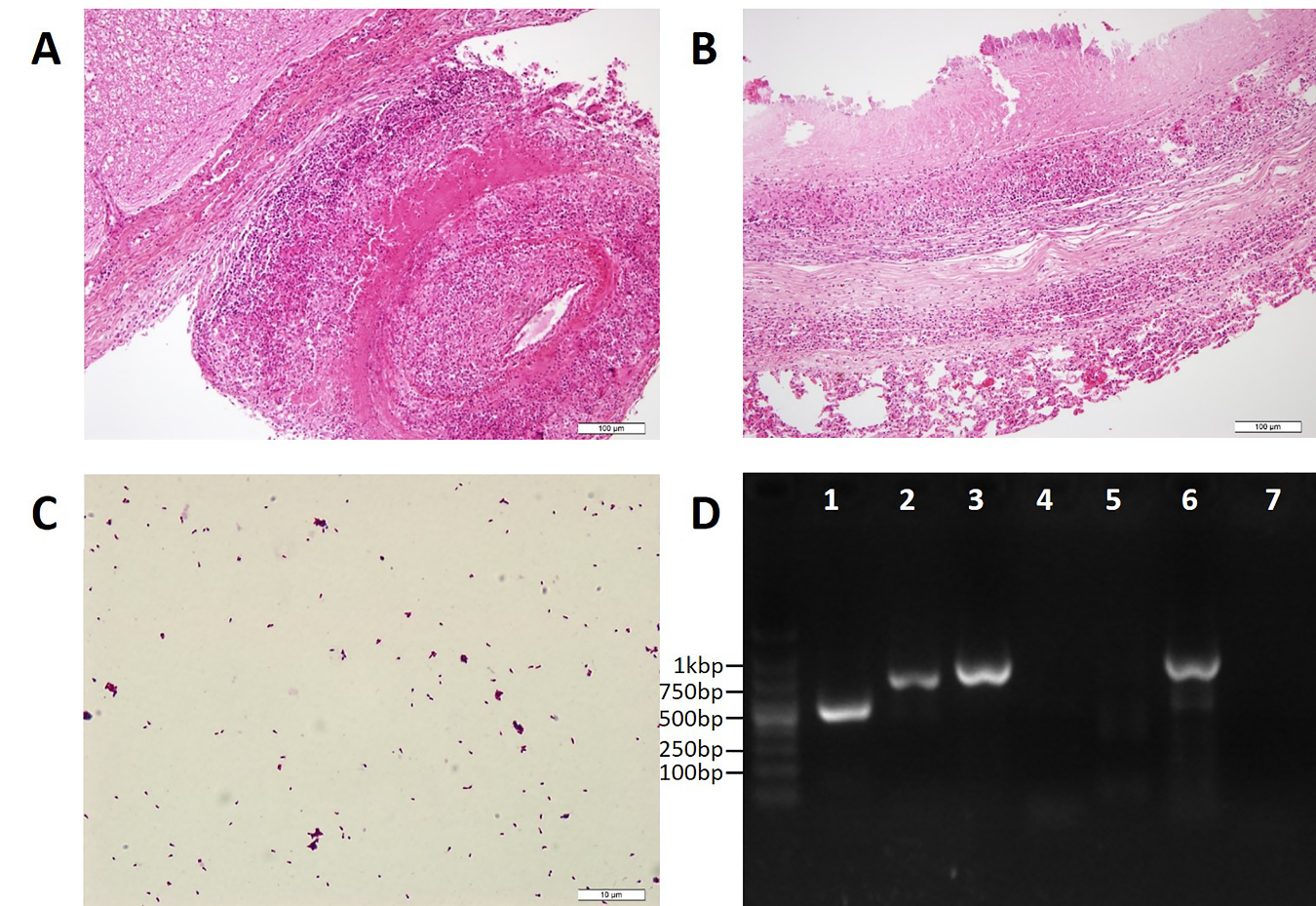
**

**S**upplementary Fig. 1. The identification of newly isolated *M. bovis* N. (A) A classic meningeal nodular granuloma with central necrotic area. (B) A granulomatous lesion in the lung, we could see alveolar tissue aside the connective tissue layer. (C) Ziehl staining, identification of the isolated strain. (D) The composite MTBC PCR typing of the isolated strain. Lanes: 1, 16S rRNA; 2, Rv0577; 3, IS1561"; 4, Rv1510; 5, Rv1970; 6, Rv3877/8; 7, Rv3120. Scale bar: 100 μm (A and B), 10 μm (C).


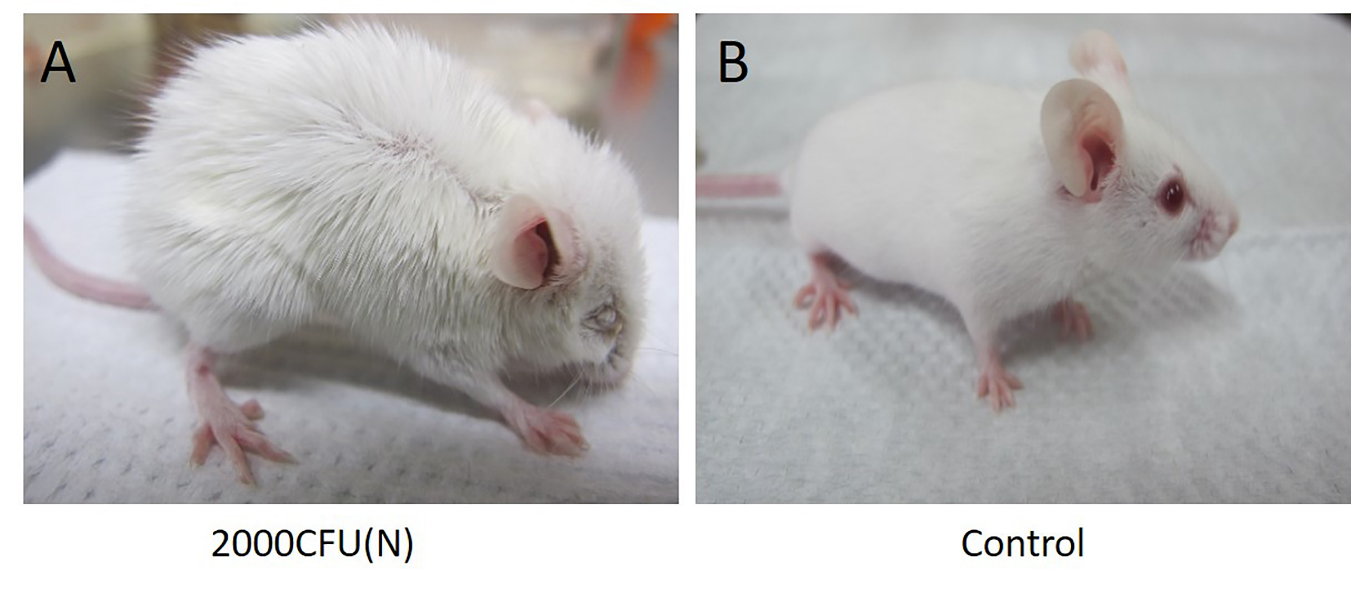


**Supplement Fig.** 2. The representative photo of the mice with typical clinical symptoms. (A) The mouse inoculated with 2000 CFU *M. bovis* N when sacrificed (30 days post inoculation). (B) The mouse inoculated with PBS.


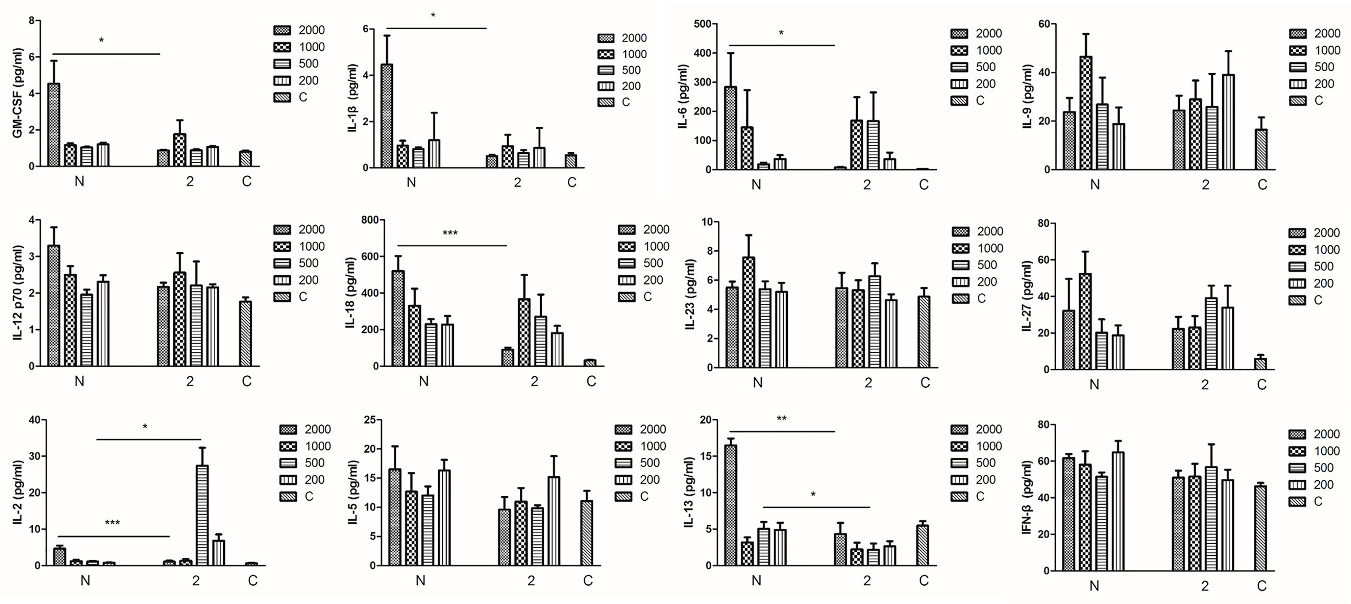


**Supplement Fig.** 3. Cytokines in serum from mice infected with different concentrations of strains *M. bovis* N and *M. bovis* C68004. The blood was harvested ante-mortem via eye route. Cytokines shown are GM-CSF, IL-1β, IL-6, IL-9, IL-12, IL-18, IL-23, IL-27, IL-2, IL-5, IL-13, IL-β. Statistical analysis was performed using student’s unpaired t-test (two-tailed). Bars represent means SEM (n=6); *P < 0.05, **P < 0.01; ***P < 0.001.

**S**upplementary Table. 1. Indels (insertions/deletions) of Genomic sequence analysis. (Sheet 1) All Indels existed in strains of *M. bovis* N and *M. bovis* C68004 and compared with the reference strain of *M. bovis* AF2122 (ATCC##). (Sheet 2) The Indels which were corresponding to the VFDB database.

**S**upplementary Table. 2. SNPs (single nucleotide polymorphisms) of Genomic sequence analysis. (Sheet 1) All SNPs existed in strains of *M. bovis* N and *M. bovis* C68004 and compared with the reference strain of *M. bovis* AF2122 (ATCC##). The SNPs which were corresponding to the VFDB database.
